# Examination of risk factors for discontinuation of follow‐up care in patients with head and neck cancer

**DOI:** 10.1002/cam4.4944

**Published:** 2022-06-12

**Authors:** M. Bryant Howren, Alan J. Christensen, Nitin A. Pagedar

**Affiliations:** ^1^ Department of Behavioral Sciences & Social Medicine, College of Medicine Florida State University Tallahassee Florida USA; ^2^ Florida Blue Center for Rural Health Research & Policy, College of Medicine Florida State University Tallahassee Florida USA; ^3^ Department of Psychology East Carolina University Greenville North Carolina USA; ^4^ Department of Otolaryngology—Head and Neck Surgery, Carver College of Medicine The University of Iowa Iowa City Iowa USA

**Keywords:** cancer survivorship, depressive symptoms, discontinuation of care, head and neck cancer, retention in care

## Abstract

**Background:**

Little research has examined discontinuation of follow‐up care in patients with head and neck cancer. This exploratory study sought to examine key demographic, disease, and behavioral factors as possible correlates of discontinuation (*N* = 512).

**Methods:**

Cross‐sectional study examined correlates of discontinuation of follow‐up care within 1 year. The primary outcome was defined as a disease‐free survivor not returning to cancer clinic for two consecutive follow‐up appointments within the first year of care and not reentering oncologic care at any point thereafter. Demographic, disease, and behavioral factors were examined using multivariable logistic regression.

**Results:**

One hundred twenty‐six (24.6%) patients discontinued by 12‐month follow‐up. Being unmarried (OR = 1.28, 95% CI = 1.01–1.63, *p* = 0.041) and having elevated depressive symptomatology (OR = 1.04, 95% CI = 1.01–1.07, *p* = 0.034) were significantly associated with discontinuation. Receipt of a single (vs. multimodal) treatment approached significance (OR = 1.71, 95% CI = 0.96–3.07, *p* = 0.071).

**Conclusion:**

Approximately one quarter of patients disengaged from important follow‐up care within 1 year. Lack of social support, depressive symptomatology, and single treatment modality may be important correlates of discontinuation of care in patients with head and neck cancer. Additional studies of this outcome are needed. Improved understanding of correlates associated with discontinuation could facilitate the identification of at‐risk patients and further development of interventions to keep patients engaged at a crucial time in the survivorship care trajectory.

## Abstract 2

This exploratory study utilized data from a longitudinal study of head and neck cancer outcomes (N=512) to examine discontinuation of follow‐up care within one year and found that approximately one quarter disengaged from care. Multivariable logistic regression was used to examine correlates of discontinuation with lack of social support, depressive symptomatology, and single treatment modality possibly associated with this behavior.

## 1 INTRODUCTION

Head and neck cancer (HNC)—which is defined as cancer of the oral cavity, pharynx (nasopharynx, oropharynx, and hypopharynx), larynx, nasal cavity, paranasal sinus, and/or salivary glands—is the seventh most common type of cancer and represents a significant worldwide burden with approximately 890,000 incident cases each year.^1^, ^2^, ^3^ Treatment for HNC, such as surgery, radiation, and chemotherapy, may lead to numerous side effects including difficulties with swallowing, breathing, eating, and speech.^2^, ^3^ These side effects can lead to disruption of essential daily functions, psychological distress, and diminished health‐related quality of life (HRQOL).^4^

Historically, the success of cancer treatment has largely been measured in terms of survival, but as more patients with a HNC diagnosis continue to live longer, focus on survivorship care beyond the treatment phase is important.^5^ When treatment sequelae are coupled with the risk for HNC recurrence and second primary cancers,^3^ the time soon after treatment represents a vital period which depends on continued, effective follow‐up care into the survivorship period to achieve optimal HNC‐specific outcomes. This is particularly true during the first year of the survivorship trajectory when side effects of treatment may be most obvious and risk of recurrence is increased.^3^, ^6^, ^7^ However, a nontrivial number of patients may discontinue care soon after treatment, putting them at risk of negative outcomes.^8^, ^9^

Some existing work has examined factors predicting utilization of follow‐up cancer care in HNC survivors which is a related but conceptually distinct phenomenon from discontinuation.^10^, ^11^, ^12^ These studies have suggested that patient comorbidity, treatment modality, and geographic location among others are associated with continued engagement in follow‐up care. While exploration of these factors is needed, research also demonstrates that utilization of follow‐up care declines with time.^10^, ^12^ One recent study found that only half of patients receive recommended surveillance testing over a 2‐year period posttreatment, for example.^12^ Limited research to date suggests that factors such as marital status, treatment modality, and poor recognition of the recommended follow‐up period may be related to discontinuation in HNC,^8^, ^9^ but overall extremely little is known about those who discontinue follow‐up care. This includes rates of discontinuation and which key demographic, disease, and behavioral factors may play a role, thus warranting further investigation.

The present exploratory cross‐sectional substudy derived from a larger parent study of oncologic treatment outcomes therefore examines potential correlates, assessed at diagnosis, of discontinuation within the first year of follow‐up care in 562 patients with HNC. Although survivorship care utilization has been the subject of numerous studies, we as yet know little about factors that may be associated with disengagement from follow‐up care in HNC. In addition to common demographic and disease characteristics including age, cancer site and stage, marital status, race, sex, and treatment modality—which were collected via self‐report or abstracted from the patient's medical record, as appropriate—this study examined whether problem alcohol use, tobacco use, depressive symptomatology, and rurality were associated with discontinuation of follow‐up care. Problem alcohol use, tobacco use, and depressive symptomatology have been shown to be related to negative outcomes in HNC during the first year of care^4^, ^13^, ^14^ and rurality may represent a barrier to continued follow‐up care due to distance to clinic. Better understanding of discontinuation in patients with HNC may help inform efforts to identify at‐risk patients, improve follow‐up care delivery, and reduce care disparities during a period soon after treatment in which follow‐up care is needed for continuous recovery and good HRQOL.^6^

## 2 METHODS

### 2.1 Participants and procedure

Participants were sampled from of a larger longitudinal study of oncologic treatment outcomes in HNC in which individuals ≥18 years old with upper aerodigestive tract carcinomas from the Department of Otolaryngology's head and neck oncology clinic at The University of Iowa Hospitals and Clinics (UIHC) were eligible. These patients were enrolled in the Outcomes Assessment Project (OAP) which successfully recruited approximately 76% of all eligible patients with HNC seen at UIHC from November 1998 through December 2014, the period including the sample of patients described below. Patients' site and stage of cancer, comorbidities, treatment, disease and survival outcome, demographics (age, race, and sex), and other clinical and psychosocial information were collected as part of the OAP via self‐report or abstracted from the patient's medical record, as appropriate. At the time of diagnosis, patients were offered participation in a longitudinal study of cancer‐related outcomes and consented in writing if interested in participating in the study. Demographic, disease, and treatment information were collected at this time. Based on UIHC care team recommendations, patients are seen at 3‐month intervals after diagnosis (i.e., 3‐, 6‐, 9‐, and 12‐months) during the first year of treatment and follow‐up. Research assessments corresponded with these follow‐up care intervals in OAP. Subsequent research assessments were completed at regular intervals beyond 12‐months, beginning annually through year 5, then at year 10 and 15 if applicable. All study procedures were approved by The University of Iowa's IRB (#199412746).

### 2.2 Measurement of key variables

#### 2.2.1 Discontinuation of care

Discontinuation of follow‐up care was operationally defined as having occurred if a survivor, determined to be cancer‐free at the conclusion of treatment, did not return to a UIHC cancer clinic for two consecutive follow‐up appointments within the first year of care and did not reenter oncologic care at any subsequent time point during the first year or beyond. For example, a patient with appointment records at 3‐ and 6‐months but not at 9‐ and 12‐months (with no subsequent clinical encounters) would be considered discontinued. A patient missing only the 12‐month follow‐up appointment within the first year would not be considered discontinued for this study. This definition is similar to that used in another recent study of discontinuation of care in HNC.^9^ Patients deceased at 365 days were excluded (*N* = 50; 8.9%) and not considered part of the analytic sample for this study, therefore leaving a final *N* of 512; survival information was abstracted from the patient's medical record. Patients were considered cancer‐free if a cancer‐free status after curative‐intent treatment was confirmed during at least one visit approximately 90 days after treatment completion.

#### 2.2.2 Problem alcohol use

The Short Michigan Alcoholism Screening Test is a self‐report screening tool designed to detect problem drinking and alcohol use disorder.^15^ The SMAST has been used in numerous patient populations, including previous studies of patients with HNC.^16^ Items include, “*Do you feel that you are a normal drinker*?” and “*Are you able to stop drinking when you want to?*” Adequate reliability and validity have been reported.^17^ Items are presented in yes/no format, with scores ranging from 0 to 13. A score of 3 or higher suggests probable alcohol abuse.^15^, ^17^ The SMAST was collected at parent study enrollment, which was used to create a dichotomous variable indicating problem use (vs. not).

#### 2.2.3 Depressive symptoms

The Beck Depression Inventory (BDI) was used to assess depressive symptoms. The BDI is a widely used and well‐validated measure of depressive symptomatology consisting of 21 items scored 0–3, each assessing a unique category of depressive symptoms.^18^ Cutoff scores have been established which suggest minimal (0–9), mild (10–18), moderate (19–29), and severe (30–63) depression.^18^ The BDI has been used in both nonclinical and clinical samples, including those with HNC.^19^ Scores were treated continuously in multivariable logistic regression in this study.

#### 2.2.4 Rurality

Because rurality is a factor known to be associated with access to care, it was deemed an important variable in this exploratory study. Rurality was determined using the US Department of Agriculture's Rural Urban Commuting Area (RUCA) codes. RUCA utilizes a 10‐point classification system and includes primary commuting flow and secondary commuting flow scores, based on the 2010 census data. Based on the recommendations of the University of Washington's Rural Health Research Center two category classification system, categorization C,^20^ rural codes were 4.0, 4.2, 5.0, 5.2, 6.0, 6.1, 7.0, 7.2, 7.3, 7.4, 8.0, 8.2, 8.3, 8.4, 9.0, 9.1, 9.2, 10.0, 10.2, 10.3, 10.4, 10.5, and 10.6. Patient addresses were abstracted from research records to establish RUCA codes.

#### 2.2.5 Other key variables

Demographic and behavioral variables including age, marital status, race, sex, and tobacco use (current/previous/never) were collected via self‐report at the initial research assessment upon parent study enrollment. Cancer site, stage, and treatment modality were abstracted from the patient medical record. Cancer site was categorized as oral cavity, oropharynx, hypopharynx, larynx, or other and cancer stage using the American Joint Committee on Cancer (AJCC) classification, stages 0‐IV. As the sample comprises patients diagnosed and enrolled from 1998 through 2014, the 5th, 6th, and 7th AJCC editions were used for staging depending on the edition in effect at the time of diagnosis. Treatment modality was dichotomized as single (chemotherapy or radiotherapy or surgery) versus multimodal.

### 2.3 Statistical analyses

Multivariable logistic regression analysis was used to examine key demographic, disease, and behavioral factors, assessed at diagnosis, as potential predictors of discontinuation within the first year of follow‐up care. Variables entered into the model were age, cancer site and stage, depressive symptoms, marital status, problem alcohol use, race, rurality, sex, treatment modality (single vs. multimodal), and tobacco use. Analyses were conducted using SPSS, version 27. Results were considered statistically significant if *p* < 0.05. Odds ratios, 95% confidence intervals, and *p*‐values for Wald chi‐squared test for logistic regression are reported below.

## 3 RESULTS

Table 1 summarizes the demographic, disease, and behavioral characteristics of the study sample (*N* = 512). Those patients who discontinued care by 12‐month follow‐up (*N* = 126, 24.6%) had a mean age of 61.1 (*SD* = 12.3) which was similar to those patients who continued care through the first year (*N* = 386, 75.4%; *M* = 59.7, *SD* = 12.0). The majority of patients in both groups were married/living with partner (61.1% vs. 66.1%), diagnosed with advanced disease stage (i.e., III or IV; 51.6% vs. 53.6%), and the most common site in both groups was oral cavity (42.8% vs. 40.2%). The percentage of patients scoring 3+ on the SMAST suggesting problem alcohol use was 21.4% for the discontinued group versus 16.1% for patients who continued care. The mean BDI score was slightly higher in the discontinued group (*M* = 9.36, *SD* = 7.55 vs. 7.94, *SD* = 7.60), with a greater percentage scoring in the range of at least mild depressive symptomatology. Approximately 45% of each group was classified as rural.

**TABLE 1:** Patient, disease, and treatment characteristics at diagnosis

	Discontinued *N* = 126 (24.6%)	Continued *N* = 386 (75.4%)	*p*
Age			0.26
Mean (*SD*); range	61.1 (12.3); 32–90	59.7 (12.0); 25–91	
Marital status			0.31
Married/living with partner	77 (61.1%)	255 (66.1%)	
Unmarried/divorced/widowed	49 (38.9%)	131 (33.9%)	
Race			0.79
Black/Other	7 (5.6%)	24 (6.2%)	
White	119 (94.4%)	362 (93.8%)	
Rurality			0.93
Rural	57 (45.2%)	173 (44.8%)	
Urban	69 (54.8%)	213 (55.2%)	
Sex			0.09
Male	88 (69.8%)	237 (61.4%)	
Female	38 (30.2%)	149 (38.6%)	
Site			0.91
Oral cavity	54 (42.8%)	155 (40.2%)	
Oropharynx	25 (19.8%)	85 (22.0%)	
Hypopharynx	6 (4.8%)	13 (3.4%)	
Larynx	22 (17.5%)	73 (18.9%)	
Other	19 (15.1%)	60 (15.5%)	
Stage			0.69
Early (0–II)	61 (48.4%)	179 (46.4%)	
Advanced (III–IV)	65 (51.6%)	207 (53.6%)	
Treatment			0.02
Single modality (chemotherapy/radiotherapy/surgery)	82 (65.1%)	204 (52.8%)	
Multimodality	44 (34.9%)	182 (47.1%)	
Alcohol use			0.17
Problem use (SMAST 3+)	27 (21.4%)	62 (16.1%)	
No/nonproblem use	99 (78.5%)	324 (83.9%)	
Tobacco use			0.09
Current	44 (34.9%)	97 (25.1%)	
Previous	54 (42.8%)	181 (46.9%)	
Never	28 (22.2%)	108 (27.9%)	
BDI score (Mean/*SD*); range	9.36 (7.55); 0–46	7.94 (7.60); 0–46	0.07
Minimal (0–9)	74 (58.7%)	268 (69.4%)	0.06
Mild (10–18)	44 (34.9%)	96 (24.9%)	
Moderate (19–29)	4 (3.2%)	17 (4.4%)	
Severe (30+)	4 (3.2%)	5 (1.3%)	

*Note*: *T*‐test and chi‐square test used as appropriate.

Abbreviations: BDI, Beck Depression Inventory; SMAST, Short Michigan Alcoholism Screening Test.

Multivariable logistic regression analysis predicting discontinuation of care by 12‐month follow‐up is presented in Table 2. Significantly increased odds of discontinuation were observed for those who were unmarried/divorced/widowed (OR = 1.28, 95% CI = 1.01–1.63, *p* = 0.041) and for those with elevated depressive symptoms at diagnosis (OR = 1.04, 95% CI = 1.01–1.07, *p* = 0.034). Having received single (vs. multimodal) treatment approached significance (OR = 1.71, 95% CI = 0.96–3.07, *p* = 0.071). Age, cancer site and stage, problem alcohol use, race, rurality, sex, and tobacco use all were not associated with significantly increased odds of discontinuation of care.

**TABLE 2:** Multivariable logistic regression analysis predicting discontinuation of care by 12‐month follow‐up

Variable	Odds ratio	95% CI	*p*‐value
Age	1.01	0.99–1.03	0.441
Marital status			
Unmarried/divorced/widowed	1.28	1.01–1.63	0.041
Married/partnered	Ref		
Race			
Black/Other	0.68	0.25–1.86	0.450
White	Ref		
Rurality			
Rural	1.13	0.67–1.89	0.646
Urban	Ref		
Sex			
Male	1.40	0.80–2.46	0.237
Female	Ref		
Site			
Oral cavity	1.65	0.64–4.26	0.301
Oropharynx	1.16	0.42–3.23	0.774
Hypopharynx	2.33	0.61–8.96	0.219
Larynx	1.05	0.37–2.94	0.933
Other	Ref		
Stage			
0–II	1.09	0.87–1.36	0.459
III–IV	Ref		
Treatment			
Single modality	1.71	0.96–3.07	0.071
Multimodality	Ref		
Alcohol use			
Problem use (SMAST 3+)	1.41	0.75–2.65	0.288
No/nonproblem use	Ref		
Depressive symptoms	1.04	1.01–1.07	0.034
Tobacco use			
Current	1.37	0.75–2.49	0.302
Previous/Never	Ref		

*Note*: *p* values correspond to the Wald chi‐squared test for logistic regression.

Abbreviation: SMAST, Short Michigan Alcoholism Screening Test.

## 4 DISCUSSION

The present exploratory study examining potential correlates of discontinuation of follow‐up care in patients with head and neck cancer found that those patients who were unmarried/divorced/widowed and those with elevated depressive symptomatology were more likely to discontinue within 1 year of entering care. Having experienced a single treatment modality approached significance (*p* = 0.071) and should also be considered in future studies. To date, very little is known about individuals who discontinue care as it is difficult to ascertain once a patient has been lost to follow‐up. Although the effects presented here are modest, the current study is important in that it examines correlates measured around the time of diagnosis which may shed some light on those most vulnerable to discontinuation posttreatment. It also provides some information regarding prevalence of discontinuation. In this sample, nearly one quarter of patients discontinued care by 12‐month follow‐up. In another study of discontinuation of care over a 3‐year period,^8^ mean time to discontinuation was 15 months and approximately one quarter disengaged. This has implications for surveillance given that the risk of HNC recurrence is elevated in the first 2 years after initial diagnosis.^21^ To date, few studies have specifically examined factors associated with discontinuation of care in HNC.^8^, ^9^

As numerous studies have demonstrated, social support is an important consideration for patients during the early survivorship period and also appears so in this context. Social support—measured by proxy in this study using marital status—has been associated with numerous outcomes related to psychosocial adjustment and survival in patients with various types of cancer, including HNC.^22^, ^23^, ^24^, ^25^, ^26^ Given the possibility of disrupted daily functioning, particularly issues with speech and communication and the potential for altered appearance, adequate social support may be especially important during the initial months after diagnosis. Some studies have also shown that single marital status may play a role in care decisions,^27^, ^28^ which has implications for discontinuation. For example, in a retrospective study of 829 patients with head and neck squamous cell carcinoma, Dronkers and colleagues^28^ examined factors associated with the declining of standard curative treatment in favor of nonstandard approaches. In multivariable analyses, they found that single marital status was significantly related to choosing a nonstandard treatment approach. As the typical HNC patient profile is majority male and over age 50,^29^ consideration should be given regarding how structured support resources may be provided to facilitate continued engagement in follow‐up care.

Depressive symptomatology may also be a contributing factor for discontinuation of care. Depression has been shown to be associated with treatment adherence and survival in patients with cancer^30^, ^31^, ^32^ and studies in HNC have demonstrated that even subclinical depressive symptomatology present before treatment initiation is associated with decreased adherence and deficits in HNC‐specific HRQOL later on.^13^, ^32^ For example, in a study examining preoperative depressive symptom severity as a predictor of postoperative functional status and adherence to adjuvant therapy, Barber and colleagues found that self‐reported moderate–severe (vs. normal‐mild) preoperative depressive symptoms were associated with lower completion of therapy.^32^ It is noteworthy that a higher percentage of discontinued patients in this study reported at least mild depressive symptomatology at diagnosis compared to those continuing treatment. Specifically, approximately 40% of patients who discontinued care reported depressive symptoms at diagnosis in the mild, moderate, or severe ranges compared to approximately 30% in the group who continued care. As the time around completion of treatment may be associated with increased depression and poor HRQOL,^6^, ^33^, ^34^ it is possible that patient depression present at diagnosis remained steady or worsened over time and may have contributed to disengagement in some cases but is in need of more complex analyses to examine this time‐dependent relationship. These findings also further underscore the need for mental health screening during this period, as per evidence‐based guidelines regarding the management of depression in cancer.^35^

Although not statistically significant at *p* < 0.05, single treatment modality approached significance (*p* = 0.071) as a predictor of discontinuation of care and deserves further attention in future studies. As cited above,^8^ a previous study of discontinuation of care in a cohort of HNC survivors over a 3‐year period (*N* = 449) found that having received a single treatment modality was significantly associated with discontinuation of care. Patients experiencing a single treatment modality may have less severe disease and fewer treatment side effects which may explain an increased likelihood of disengagement. Other studies of patient engagement in follow‐up care support this notion. For example, in one study of nearly four thousand patients with HNC, Brennan et al.^10^ used administrative data to characterize factors associated with the use of follow‐up care over 5 years posttreatment in Canada. The authors reported that having experienced multimodal treatment was associated with receiving recommended follow‐up care, including imaging tests. In another study of 100 patients with HNC in an urban, tertiary clinic it was found that those who underwent multimodal treatment were more likely to prefer increased follow‐up appointments which also meshes with the present findings.^36^ Although cancer stage may be related to receipt of single versus multimodal treatment, we did not find a relationship between cancer stage and discontinuation in this study.

Notably, being classified as rural‐residing was not associated with discontinuation of care in this sample. Some previous studies have demonstrated an association between residing in a rural area and reduced access to cancer treatment and follow‐up care, but findings are not entirely consistent.^37^, ^38^ Charlton et al.^37^ highlight many of these studies, a number of which demonstrate an association using distance as a marker of rurality. Seaman et al.^8^ examined both rurality and distance in their study and found that distance (i.e., greater than 100 miles to access point)—rather than rural classification—was associated with discontinuation. As they describe, because comprehensive cancer care is often centralized, travel distance may actually be greater for some patients classified as urban compared to rural. Similarly, in a study of 332 patients with HNC who had completed treatment and follow‐up care, Deutschmann and colleagues^11^ reported that living less than 200 miles from the treating medical center was associated with an increased likelihood of remaining engaged in follow‐up care.

Other studies examining distance and rurality in patients with cancer draw similar conclusions.^39^, ^40^ For example, in another review of studies examining the relationship between distance and several cancer‐related outcomes, it was found that patients with greater travel distance were more likely to present with advanced cancer stage and less likely to receive appropriate treatment, suggesting that distance may in part explain why patients in rural settings often fare worse on such outcomes.^41^ Distance may also confer barriers such as missing work which could result in a patient disengaging from follow‐up care. Not only should future research examine distance as it has considerable implications for access to care, but it is likely that multiple factors related to distance and rurality may interact and are relevant and deserving of additional study.^42^ Moreover, as the use of telehealth has expanded due to the COVID‐19 pandemic, investigations of telehealth delivery to mitigate distance barriers for elements of HNC follow‐up care are needed.^43^

The present study included a large sample and high accrual rate of eligible patients but is limited in several ways. First, the study sample was majority White (i.e., 90%+) and included patients from a single Midwestern healthcare system, making generalizability an issue. Second, although the larger parent study was prospective and longitudinal in its design, this cross‐sectional exploratory substudy only classified discontinuation of care within a narrow window in time. This was determined a priori, however, given the importance of the first year of treatment and follow‐up, thus making for an opportune time in the survivorship care trajectory to examine discontinuation. Third, we have no information about whether patients relocated care to another facility and thus there may be some who were misclassified as discontinued as a result. Some patients may continue follow‐up using a primary care provider closer to home, for example, rather than continuing to see a cancer care specialist. This study found a similar rate of discontinuation to Seaman et al.^8^ and although some patients may have discontinued only to engage elsewhere,^9^ such behavior still has implications for general care continuity and receipt of recommended services making identification of these patients important. Fourth, although the present study contains information regarding rural–urban status, deidentified data did not allow us to retrospectively map distance to treatment facility as zip codes were previously stripped from the data set. As discussed above, distance rather than rurality may be important in the context of discontinuation and should be modeled in future studies. Finally, some studies have suggested other psychological factors such as fear of cancer recurrence (FCR) are related to negative outcomes in HNC.^44^, ^45^ We do not have information about FCR in this study but the question of whether FCR may also be an important consideration and is an avenue for future research.

## 5 CONCLUSION

In conclusion, the results of this study suggest that upwards of one quarter of patients with HNC may discontinue important follow‐up care within the first year, which represents an important period given known deleterious physical and psychosocial outcomes during this time. In this sample, being unmarried/without a partner and having elevated depressive symptomatology were associated with discontinuation; single treatment modality, although not statistically significant, may also be important. Improved understanding of correlates associated with discontinuation could facilitate the identification of at‐risk patients and further development of interventions to keep patients educated and engaged in care at a time when it may be needed most, ensuring the needs of all HNC survivors are met over the course of the survivorship care trajectory.

## AUTHOR CONTRIBUTIONS

**M. Bryant Howren**—conceptualization, data curation, methodology, investigation, formal analysis, writing–original draft, and writing–review and editing. **Alan J. Christensen**—conceptualization, methodology, formal analysis, writing–original draft, and writing–review and editing. **Nitin A. Pagedar**—conceptualization, funding acquisition, data curation, methodology, investigation, formal analysis, and writing–review and editing.

## FUNDING INFORMATION

This work was supported in part by the National Cancer Institute and University of Iowa Holden Comprehensive Cancer Center (Award #P30 CA086862).

## CONFLICT OF INTEREST

The authors have no relevant financial or nonfinancial interests to disclose.

## ETHICS STATEMENT

This study was approved by The University of Iowa's IRB (#199412746). Written informed consent was obtained from all participants included in the study.

## Data Availability

The data that support the findings of this article are available from the corresponding author upon reasonable request.
